# Using precision mapping of schistosomiasis to guide female genital schistosomiasis (FGS) screening in Cameroon, sub-Saharan Africa

**DOI:** 10.1017/S003118202510111X

**Published:** 2025-12

**Authors:** Louis-Albert Tchuem Tchuenté, Nestor Feussom Gipwe, Emmanuelle Yimgoua, Vanessa Christinet, Jutta Reinhard-Rupp, J. Russell Stothard

**Affiliations:** 1 Centre for Schistosomiasis and Parasitology, University of Yaoundé I, Yaoundé, Cameroon; 2 Ministry of Public Health, Yaoundé, Cameroon; 3 ASCRES, Association de Soutien aux Centres de Recherches, d’Enseignements et de Soins, Geneva, Switzerland; 4 Foundation for Innovative New Diagnostics (FIND), Geneva, Switzerland; 5 Department of Tropical Disease Biology, Liverpool School of Tropical Medicine, Liverpool, UK

**Keywords:** Cameroon, female genital schistosomiasis, geospatial analysis, precision mapping, schistosomiasis

## Abstract

Schistosomiasis remains a significant public health concern in sub-Saharan Africa, particularly among women and children. In Cameroon, urogenital and intestinal schistosomiasis affect the lives of millions of impoverished populations, and female genital schistosomiasis (FGS) remains a serious threat which has not been quantified explicitly. The extent of stigmatization and discrimination related to FGS is currently unknown. This study explores the use of precision mapping to identify high-risk communities for urogenital schistosomiasis and guide targeted screening for FGS. Parasitological surveys were conducted between November 2020 and July 2021 in four health districts using urine filtration and Kato-Katz techniques, first in schools to identify areas of higher transmission, and secondly in selected high-risk communities. Geographic information system tools were employed to identify high transmission foci and households of targeted infected women. Results of surveys in schools showed no schistosomiasis transmission in Ayos (0%) and low prevalence in Akonolinga (8%), while Bertoua and Doume had high prevalence, up to 33% and 48% infection with *Schistosoma haematobium*, respectively. These results made the two health districts of Bertoua and Doume suitable for focused FGS investigations. Surveys in communities revealed higher schistosomiasis prevalence and infection intensity in Doume compared to Bertoua. Precision mapping effectively identified infected women and enabled targeted recruitment for further clinical studies, facilitating efficient resource allocation for gynaecological follow-up. This approach demonstrates the value of geospatial tools in enhancing targeted public health interventions, disease surveillance and control strategies.

## Graphical abstract

**Figure:**
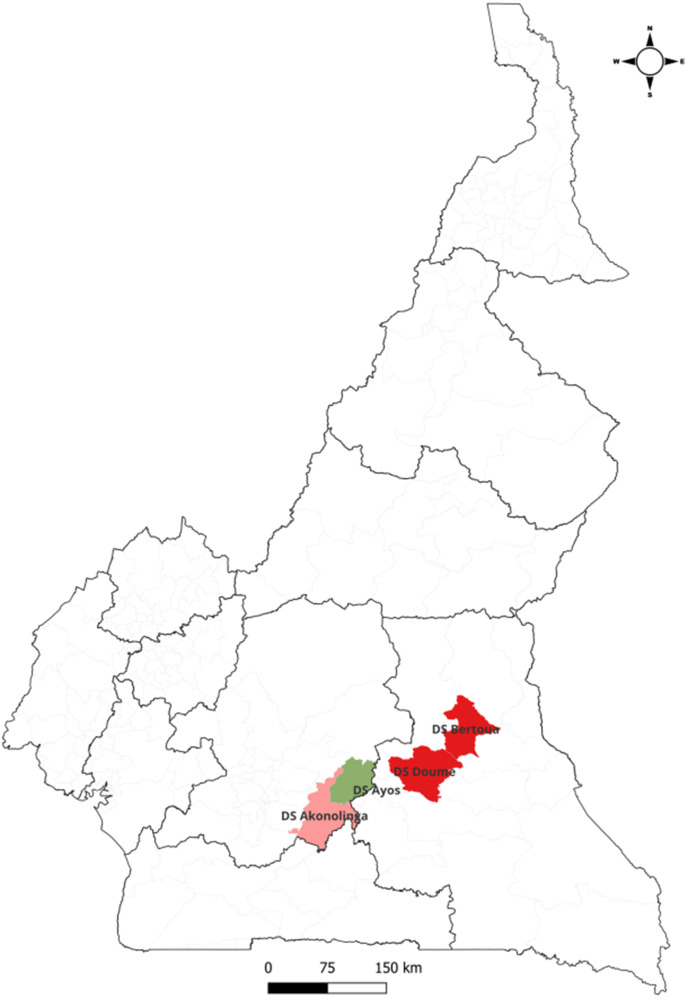

## Introduction

Schistosomiasis is a neglected tropical disease caused by parasitic trematodes of the genus *Schistosoma*, affecting over 240 million people globally (WHO 2025). Among the six species that infect humans, *Schistosoma haematobium* is responsible for urogenital schistosomiasis, which disproportionately affects women and girls, leading to female genital schistosomiasis (FGS). It is associated with gynaecological symptoms, increased susceptibility to sexually transmitted infections, and adverse reproductive health outcomes (Kjetland et al. 2012; Sturt et al. 2020; Gebremedhin et al. 2025). Despite its burden, FGS remains underdiagnosed due to limited awareness and diagnostic challenges. FGS affects the same people who carry a disproportionate global burden of HIV, HPV and cervical cancer in Africa (Kjetland et al. 2012). FGS exemplifies the experiences of marginalized women and girls, who face multiple and intersecting health, sociocultural, environmental and economic challenges. An appalling statistic within the health system and at an individual level, is that very few women are provided with a clinical diagnosis and coherent management of these infections collectively, either from static or mobile health outposts. Moreover, there can be off-putting stigma within the health service provision that mitigates patient treatment seeking behaviours (Orish et al. 2022).

In Cameroon, urogenital and intestinal schistosomiasis affect the lives of millions of impoverished populations, and it is estimated that over 2 million people are infected (MINSANTE 2005). The Government is strongly engaged to overcome this disease, and since 2006 has supported preventive chemotherapy by mass drug administration of praziquantel (Tchuem Tchuenté and N’Goran 2009). Although these interventions led to a significant decrease of infection prevalence in all regions of Cameroon, the transmission of schistosomiasis remains high, and FGS remains a serious threat which has not been quantified explicitly as is any FGS-related stigma and discrimination. The extent of stigmatization and discrimination related to FGS is currently unknown.

To develop a national strategy and properly tackle the issues of FGS in Cameroon, a pilot cross sectional study was developed to assess the feasibility and acceptability of screening strategies based on gynaecological examination in settings where this is not routinely offered to women (Nyotue et al. 2025). Given the focality of schistosomiasis transmission, the variation in infection intensities and the reduced prevalence in most health districts due to annual deworming campaigns, and considering the high costs for gynaecological investigations, it was crucial to develop an innovative approach to identify high risk communities and infected women for targeted recruitment for the clinical examinations. Therefore, precision mapping of schistosomiasis was used to guide FGS screening, focusing on identifying communities and individuals with urogenital schistosomiasis. Indeed, precision mapping has emerged as a powerful tool in disease surveillance and control, enabling high-resolution identification of schistosomiasis transmission settings and guiding mass drug administration and resource allocation (Tchuem Tchuenté et al. 2018).

## Materials and methods

### Study site

**Figure 1.**
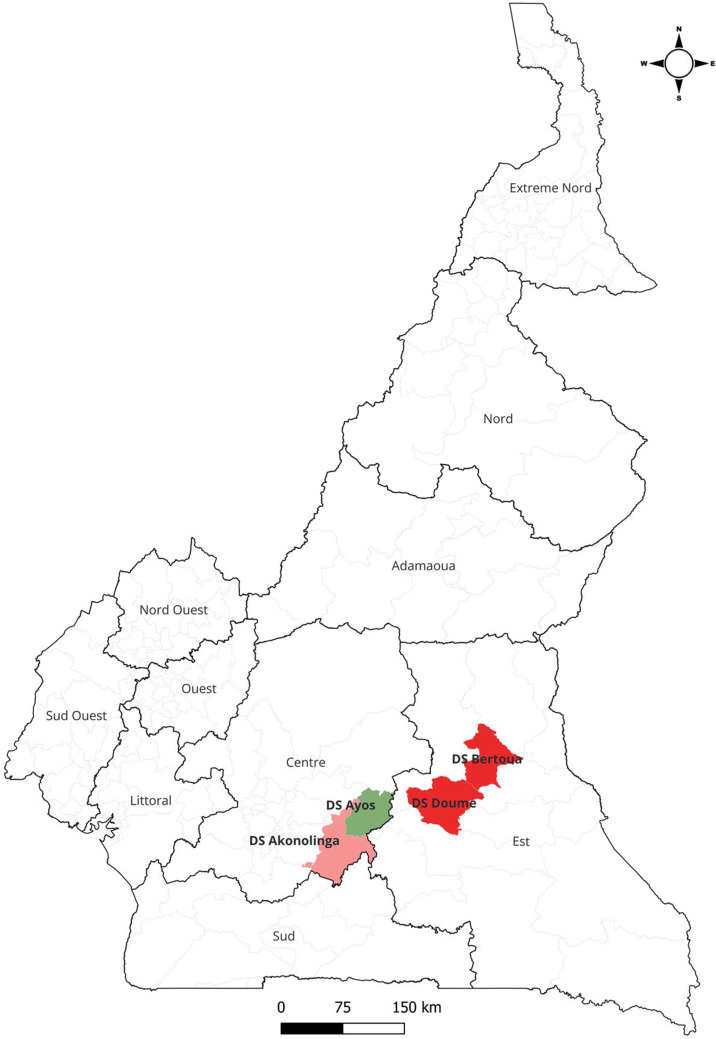
Map of Cameroon showing the locations of the four health districts included in the study: Akonolinga and Ayos in the Centre Region, and Bertoua and Doume in the East Region.

The study was conducted in four health districts, including two in the Centre Region of Cameroon, i.e. Akonolinga and Ayos, and two in the East Region, i.e. Bertoua and Doume (Figure 1). These health districts were selected for their epidemiological relevance to schistosomiasis and represent a mix of urban and rural settings. Akonolinga health district is located along the Nyong River and has a population of approximately 89 012 inhabitants. Ayos health district is situated at the confluence of the Nyong and Long-Mafog rivers and covers a broader area with a population of 44 910. Bertoua health district includes Bertoua, the capital of the East Region, a major urban centre with a population of approximately 165 000. The health district includes urban and peri-urban zones and serves as a referral hub for surrounding rural areas, with a total population of 391 174. Doume is a rural health district in the Haut-Nyong department, with a combined population of approximately 57 044. The health districts of Akonolinga, Ayos, Bertoua, and Doume are subdivided into 23, 12, 16, and 12 health areas (sub-districts), respectively (MINSANTE 2025).

### Sampling and data collection

Parasitological surveys were conducted between November 2020 and July 2021 in the health districts of Ayos, Akonolinga, Bertoua, and Doume. Sample collection was coordinated by the Centre for Schistosomiasis and Parasitology (CSP), with support from regional health and education authorities. The surveys were conducted in two phases, first in schools and secondly in communities. The surveys in schools allowed to identify the high endemic communities to be selected for the implementation of a wider community survey for the identification of infected persons and their households, who were subsequently selected for clinical and gynaecological consultations.

#### Sampling in schools

In order to assess the current levels of infections in the four-targeted health districts, the schools were selected using the list of villages and schools, taking into account the ecological zones, the risk factors and the prior local knowledge for schistosomiasis transmission. Schools were selected in all health areas (sub-districts), with a relatively even spatial coverage. In each district, schools with previously known higher prevalence or located in areas of high risk of transmission were selected in priority. The geographical coordinates of each sampled school were recorded using global positioning system (GPS) devices. In each school, 50 urine and 50 stool samples were collected from 25 boys and 25 girls. Children were preferentially selected from the 5th and 6th grades, and then from other grades where the number of children in the 5th and 6th grades was fewer than 50 (Tchuem Tchuenté et al. 2012). The samples were collected in 60 mL plastic screw-cap vials, between 10·00 AM and 2·00 PM. In addition to biological samples, demographic information – including sex, age, and residential quarter – was recorded for each child using electronic tablets.

#### Sampling in communities

Parasitological surveys conducted in schools enabled the identification of communities with the highest prevalence of urinary schistosomiasis. These high-endemic communities were subsequently selected for broader community-based surveys aimed at identifying infected individuals and their respective households. Additionally, information on children’s residential quarters, along with epidemiological risk factors such as proximity to water sources, was used to further refine the selection of areas at highest risk for schistosomiasis transmission. Prior to sample collection, initial contact was established with community leaders, heads of residential quarters, and local health personnel. They were informed about the design and objectives of the precision mapping surveys and actively participated in planning the sample collection process. Similarly, residents of the targeted communities were sensitized and briefed on the study. Their involvement was instrumental in selecting appropriate households for participation in the surveys. In each selected community, parasitological surveys were conducted through a door-to-door approach. Urine samples were collected in 60 mL plastic screw-cap vials, exclusively from women aged 13 to 50 years. In addition to biological samples, demographic data (including sex and age) and household GPS coordinates were recorded using electronic tablets. Only urine samples were collected during the community surveys.

#### Analysis of stool and urine samples

All urine and stool samples were transported to the local health district hospital laboratory and examined the same day of collection. In the laboratory, each urine sample was agitated to ensure adequate dispersal of eggs, 10 mL of urine were filtered through a Nucleopore® filter, and the filters were examined by microscopy for the presence of schistosome eggs. Stool samples were examined by a single thick smear technique using a 41·7 mg Kato-Katz template. Each slide was read twice; immediately after slide preparation for hookworm eggs, and later for schistosome and other soil-transmitted helminth (STH) eggs. Parasitic infections were recorded; number of eggs for each parasite was counted; and intensity of infection was calculated and expressed as eggs per gram of faeces (epg) or eggs per 10 mL of urine (egg/10 mL).

### Data analysis

For the precision mapping, the key indicators are the rates of infection prevalence and intensities within populations in the different districts, subdistricts and communities. Data from parasitological surveys were entered into Microsoft Excel, checked for accuracy, and imported into SPSS version 28 (IBM Corp.) for statistical analyses. Prevalence was calculated as the proportion of individuals testing positive for *Schistosoma* eggs among all individuals examined. For school-based surveys, the Complex Samples Crosstabs procedure was used to account for the cluster design, with health districts defined as strata and schools as primary sampling units. This adjustment incorporated finite population corrections under equal probability sampling without replacement. For all prevalence estimates, 95% confidence intervals (CIs) were calculated using the Wilson score method without continuity correction after weighting for the sampling design. Infection intensity was summarized as both arithmetic mean and geometric mean egg counts (eggs/10 mL for *S. haematobium*; eggs per gram of stool for *S. mansoni*). Because egg count data were highly skewed, geometric means were computed after log-transformation of positive egg counts, with 95% CIs calculated on the log scale using the t-distribution before back-transformation to the original scale. Comparisons of schistosomiasis prevalence between sexes and across age groups were performed using the Chi-square test. Differences in infection intensity between health districts were assessed using independent samples t-tests. Variation in infection intensity across multiple age groups within districts was examined using one-way analysis of variance (ANOVA) followed by pairwise comparisons when appropriate. The association between infection intensity and age was explored using Pearson correlation analysis. All statistical tests were two-sided, with a significance threshold of *P* < 0·05. Geographical coordinates of schools and households were imported into ArcGIS Pro 3.4 (ESRI Inc., USA) to generate precision maps displaying the spatial distribution of infection prevalence and intensity across the study areas.

## Results

### Prevalence and intensity of schistosomiasis in schools

A total of 125 schools were surveyed: 28 schools in the Akonolinga health district, 12 in Ayos, 27 in Bertoua and 58 in Doume. A total of 5841 pupils from these 125 schools were registered and included in the study. Of these children registered, 5713 (97·81%) and 5620 (96·22%) provided urine and stool samples, respectively.

**Figure 2.**
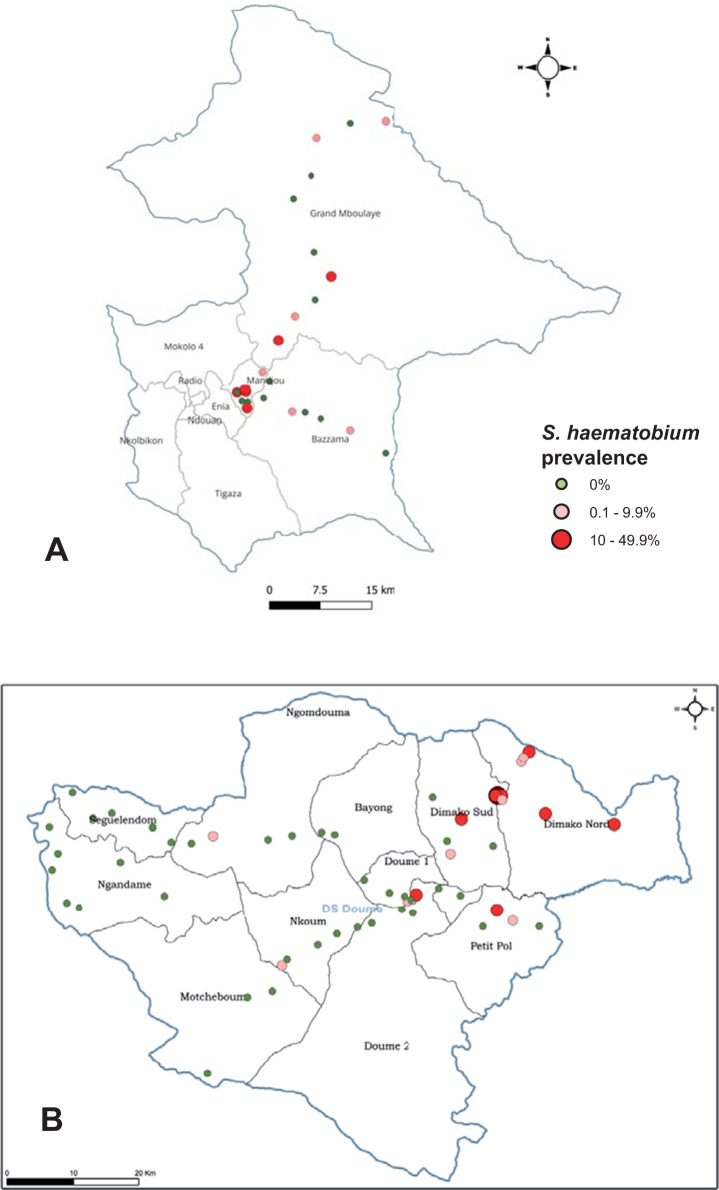
Maps of schistosomiasis prevalence in schools in the health districts of Bertoua (A) and Doume (B) in the East Region of Cameroon. The colours represent school infection levels, with green for schools where no infected child was found, pink for prevalence between 0·1 and 9·9, red for prevalence 10 and 49·9, and dark red for prevalence≥ 50. Produced with Esri ArcGIS Pro 3.4.

Table 1 summarizes the survey results for individual parasites in each health district. The results are shown as prevalence and intensity of infections together with 95% CI. *S. haematobium* infected children were found in 32 of the 125 (25·6%) schools investigated, with an average overall prevalence of 3·1% ranging from 0% to 48% across the four health districts. *S. mansoni* was found in 9 schools with an average prevalence of 0·2%, ranging from 0% to 5% across the four health districts. *S. guineensis* was not found in the four health districts. The highest prevalence of schistosomiasis, 48% for *S. haematobium*, was found in Doume health district, East region. The point prevalence distribution of schistosomiasis in all surveyed schools is shown in Figure 2. There was an evident difference of schistosome infections between surveyed schools. The majority of schools were negative for schistosomiasis. There was no significant difference of schistosomiasis prevalence between boys and girls (Chi-square test, *χ*^2^ = 0·94, *P* > 0·05) in the four health districts.

**Table 1.** Adjusted prevalence and geometric mean intensity of parasitic infections (95% CI) in school children in the health districts of Akonolinga and Ayos in the Centre Region, Bertoua and Doume in the East Region of Cameroon. Surveys conducted between November 2020 and July 2021

	No. of persons examined	*Schistosoma haematobium*	*Schistosoma guineensis*	*Schistosoma mansoni*	*Ascaris lumbricoides*	Hookworm	*Trichuris trichiura*
**Prevalence (%)**							
*Overall*	5841	3·10 (2·65−3·54)	0·00 (0·00−0·00)	0·21 (0·10−0·33)	42·97 (41·71−44·24)	3·85 (3·37−4·35)	26·95 (25·82−28·09)
*By health district*							
Akonolinga	1283	0·39 (0·05−0·73)	0·00 (0·00−0·00)	0·23 (0·00−0·50)	44·97 (42·25−47·70)	2·03 (1·26−2·80)	39·13 (36·45−41·80)
Ayos	641	0·00 (0·00−0·00)	0·00 (0·00−0·00)	0·00 (0·00−0·00)	25·12 (21·75−28·48)	0·78 (0·10–1·46)	14·98 (12·21−17·74)
Bertoua	1163	4·90 (3·66−6·14)	0·00 (0·00−0·00)	0·43 (0·05−0·81)	9·72 (8·01−11·42)	9·54 (7·85−11·23)	2·92 (1·95−3·89)
Doume	2754	4·32 (3·56−5·08)	0·00 (0·00−0·00)	0·15 (0·00−0·29)	60·24 (58·41−62·07)	3·01 (2·37−3·65)	34·20 (32·43−35·98)
*By sex*							
Male	2913	3·47 (2·80−4·13)	0·00 (0·00−0·00)	0·14 (0·00−0·27)	41·88 (40·09−43·67)	3·71 (3·02−4·39)	27·43 (25·81−29·05)
Female	2684	2·98 (2·34−3·62)	0·00 (0·00−0·00)	0·26 (0·07−0·45)	42·21 (40·34−44·08)	4·17 (3·42−4·93)	25·15 (23·51−26·79)
Intensity of infection (epg)*							
*Overall*	5841	23·34 (17·34−31·42)	0·00 (0·00−0·00)	234·61 (55·83−985·88)	5678·76 (5263·92−6126·30)	100·74 (84·72−119·79)	225·90 (209·58−243·50)
*By health district*							
Akonolinga	1283	16·00 (1·58−162·59)	0·00 (0·00−0·00)	112·14 (6·66−1888·51)	5222·92 (4414·34−6179·61)	93·40 (57·28−152·28)	266·45 (231·27−306·99)
Ayos	641	0·00 (0·00−0·00)	0·00 (0·00−0·00)	0·00 (0·00−0·00)	2897·58 (2021·63−4153·06)	48·00 (10·81−213·13)	135·44 (102·22−179·45)
Bertoua	1163	25·14 (12·82−49·28)	0·00 (0·00−0·00)	150·20 (5·00−4511·69)	1358·59 (928·90−1986·95)	128·74 (97·21−170·48)	124·86 (67·73−230·18)
Doume	2754	22·88 (16·64−31·47)	0·00 (0·00−0·00)	712·69 (18·13−28011·53)	6879·58 (6312·24−7497·92)	77·71 (62·09−97·26)	222·66 (202·96−244·29)
*By sex*							
Male	2913	22·43 (15·30−32·88)	0·00 (0·00−0·00)	949·32 (38·03−23699·50)	5392·11 (4827·68−6022·52)	107·32 (85·20−135·19)	201·50 (181·62−223·56)
Female	2684	24·54 (15·22−39·55)	0·00 (0·00−0·00)	132·40 (16·03−1093·48)	5875·68 (5251·71−6573·79)	·96·06 (73·55−125·47)	237·16 (211·09−266·44)

* *Note*: eggs per gram of faeces *for S. mansoni, A. lumbricoides, T. trichiura* and Hookworms; eggs/10 mL urine for *S. haematobium* intensity of infection

The egg counts ranged from 0 to 9400 eggs/10 mL for urinary schistosomiasis and from 0 to 20 736 eggs per gram for intestinal schistosomiasis. The arithmetic mean intensity of infection was 9·3 eggs/10 mL (95% CI: 3·95–14·59 eggs/10 mL) for *S. haematobium*, and 7·4 eggs per gram (95% CI: −2·48–17·23 eggs per gram) for *S. mansoni*. The health district of Bertoua was most heavily infected with both *S. haematobium* (19·0 eggs/10 mL) and *S. mansoni* (17·4 eggs per gram).

### Prevalence and intensity of schistosomiasis in communities

The findings from the surveys in schools showed low transmission of schistosomiasis in Akonolinga and Ayos health districts, while Bertoua and Doume had high prevalence, up to 33% and 48%, respectively. These results made the two health districts of Bertoua and Doume suitable for community survey and focused FGS investigations. A total of 1797 women were enrolled for the community surveys, including 687 women in Bertoua and 1110 women in Doume health district.

Table 2 summarizes the community survey results in the two health districts. *S. haematobium* infections were found in 195 women representing 10·85% of all women investigated, including 45 and 150 women in the Bertoua and Doume health districts, respectively. The arithmetic mean intensity of infection was 17·4 eggs/10 mL (95% CI: 9·83–24·97 eggs/10 mL) for *S. haematobium*, with maximum egg counts up to 1405 and 4311 eggs/10 mL in Bertoua and Doume health districts, respectively.

**Table 2.** Prevalence and intensity of *Schistosoma haematobium* (95% CI) in women in the health districts of Bertoua and Doume in the East Region of Cameroon· Surveys conducted between November 2020 and July 2021

	No· of women examined	No· infected	Prevalence (%)	Intensity of infection (eggs/10 mL)
**Bertoua health district**				
*Overall*	687	45	6·55 (4·7−8·4)	26·44 (15·33−45·57)
*By age group (years)*				
10–20	285	36	12·63 (8·77−16·49)	41·96 (23·89−73·71)
21–30	209	6	2·87 (0·61−5·13)	6·3 (2·51−15·82)
31–40	120	2	1·67 (0·00−3·96)	2·45 (1·65−3·64)
41–50	72	1	1·39 (0·00−4·09)	1 (–)
**Doume health district**				
*Overall*	1110	150	13·51 (11·5−15·52)	20·66 (15·18−28·11)
*By age group (years)*				
10–20	499	86	17·23 (13·92−20·55)	22·25 (14·93−33·17)
21–30	326	41	12·58 (8·98−16·18)	19·92 (10·92−36·35)
31–40	169	16	9·47 (5·05−13·88)	20·21 (6·88−59·35)
41–50	115	7	6·09 (1·72−10·46)	10·75 (2·88−40·05)
**Grand total**	1797	195	10·85 (9·41−12·29)	21·87 (16·72−28·59)

**Figure 3.**
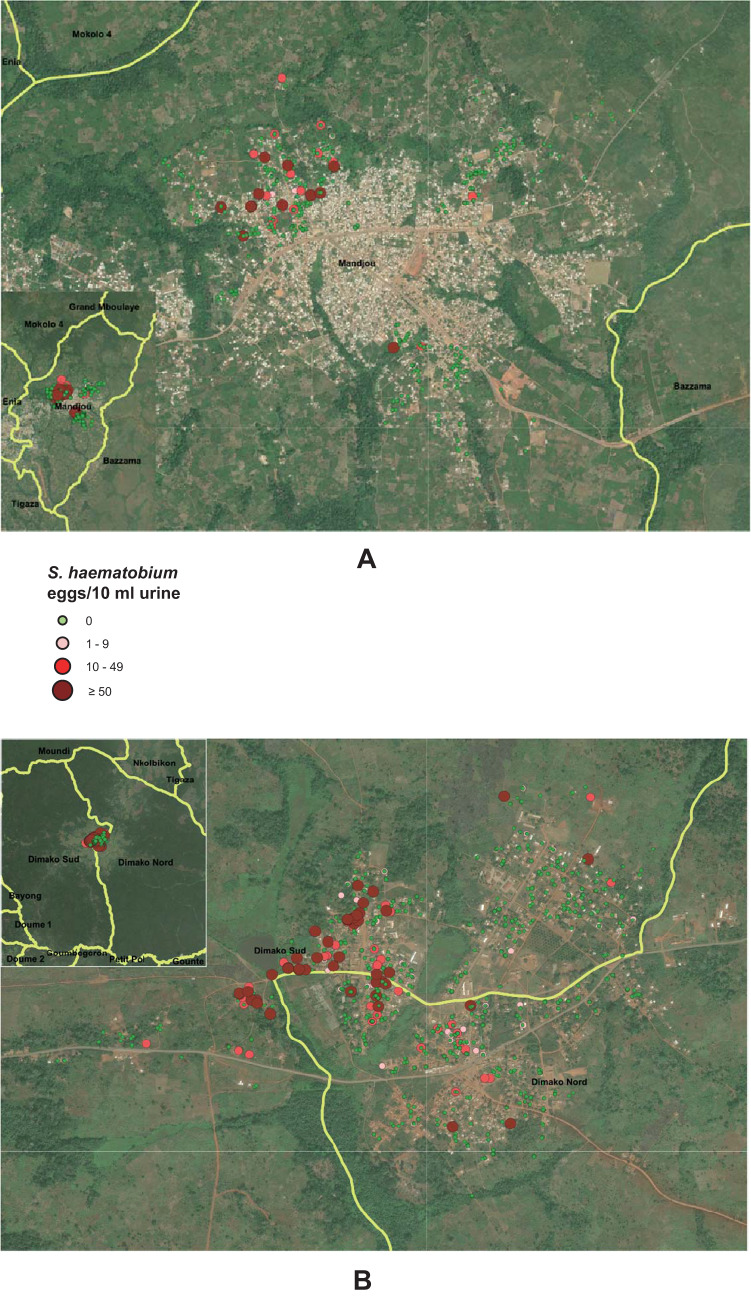
Precision maps of schistosomiasis infections in women sampled in communities in the health districts of Bertoua (A) and Doume (B) in Cameroon. The maps illustrate the household locations and individual infection levels of participants. The colours represent individual infection levels, with green for non-infected women, pink for infected women with number of eggs/10 mL between 1 and 9, red for those infected with eggs/10 mL between 10 and 49, and dark red for infected women with eggs/10 mL ≥50. Produced with Esri ArcGIS Pro 3.4.

**Figure 4.**
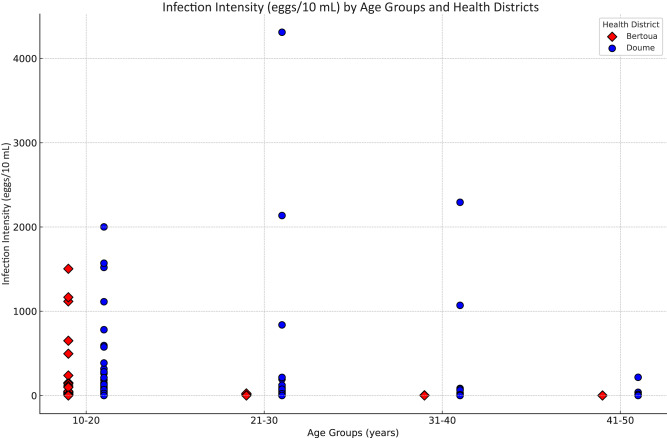
Infection intensity of schistosomiasis by age groups in the health districts of Bertoua and Doume in the East Region of Cameroon.

The household locations of women who participated in the surveys in communities are illustrated in Figure 3. The colours represent individual infection levels, with green for non-infected women, pink for infected women with number of eggs/10 mL between 1 and 9, red for those infected with eggs/10 mL between 10 and 49, and dark red for infected women with eggs/10 mL ≥ 50. The variations of infection prevalence and infection intensity by age group and health district are illustrated in Figure 4. Data analysis revealed that schistosomiasis prevalence in women decreases with age, with the highest prevalence observed in the 10–20 age group (15·56%) and the lowest in 41–50 years (4·28%). The health district of Doume consistently showed higher prevalence across all age groups compared to Bertoua, with significant differences in prevalence between districts in the 21–30 and 31–40 age groups (*P* < 0·01). The 10–20 and 41–50 groups showed no statistically significant difference, though Doume remained higher. The overall mean infection intensities was highest in 10–20 age group (23·57 eggs/10 mL) and lowest in 41–50 (1·55 eggs/10 mL), but without significant difference across age groups (ANOVA *P* = 0.3753) and weak negative correlation with age (Pearson correlation *r* = −0.0512, *P* = 0.0303). The results of the analysis by health district showed that infection intensity varied significantly by age group in Bertoua (ANOVA *P* = 0·015) whereas in Doume the variation was not significant (ANOVA *P* = 0·709). Doume exhibited significantly higher infection intensity than Bertoua (*t*-test *P* = 0·003).

### Prevalence and intensity of soil-transmitted helminthiasis in schools

Three species of STH were found in the four surveyed health districts: *Ascaris lumbricoides, Trichuris trichiura* and hookworms (Table 1). The prevalence of *A. lumbricoides* infection varied significantly across health districts. The highest prevalence was recorded in Doume (60·24%), followed by Akonolinga (44·97%), Ayos (25·12%), and Bertoua (9·72%). Geometric mean intensity also showed marked variation, with Doume exhibiting the highest intensity (6879·58 epg), followed by Akonolinga (5222·92 epg), Ayos (2897·58 epg), and Bertoua (1358·56 epg). Statistical analysis confirmed these differences. The Chi-squared test revealed a highly significant variation in prevalence across districts (*χ*^2^ = 1003·36, *P* < 0·0001). The Kruskal–Wallis test also indicated significant differences in infection intensity (*H* = 83·42, *P* < 0·0001).

Prevalence of *T. trichiura* ranged from 2·92% in Bertoua to 39·13% in Akonolinga. Doume and Ayos reported intermediate prevalence rates of 34·20% and 14·98% (96/629), respectively. Akonolinga showed the highest intensity (266·45 epg), followed by Doume (222·66 epg), and Bertoua (124·86 epg). The Chi-squared test showed significant differences in prevalence across districts (*χ*^2^ = 566·65, *P* < 0·0001). The Kruskal–Wallis test for intensity also yielded a significant result (*H* = 25·78, *P* < 0·0001). Hookworm infections were less prevalent overall but still demonstrated inter-district variation. Bertoua had the highest prevalence at 9·54%, followed by Doume at 3·01%, Akonolinga at 2·03%, and Ayos at 0·78%. The intensity values ranged from 48·00 epg in Ayos to 128·74 in Bertoua. There was a significant difference in prevalence across districts (*χ*^2^ = 136·39, *P* < 0·0001), whereas the difference in intensities was not significant (*H* = 6·67, *P* = 0·0833).

## Discussion

This study underscores the strategic value of precision mapping as a tool for identifying high-transmission areas of urogenital schistosomiasis. Our results demonstrate substantial spatial heterogeneity in the transmission of *S. haematobium* across the four surveyed health districts in Cameroon. The absence of active schistosomiasis infections in Ayos and the markedly low prevalence observed in Akonolinga reflect the positive impact of sustained national mass drug administration (MDA) campaigns with praziquantel, consistent with recent national assessments highlighting significant reductions in overall schistosomiasis burden (Tchuem Tchuenté et al. 2012, 2013; Ministry of Public Health Cameroon 2020). However, the persistently high prevalence documented in Bertoua and Doume exposes the limitations of uniform, nationwide approaches and underscores the need for geographically tailored interventions targeting residual transmission hotspots. Such localized persistence of infection is emblematic of the focal nature of schistosomiasis, as described in multiple transmission settings across sub-Saharan Africa (Kittur et al. 2019; Lim et al. 2023; Trippler et al. 2025). Boxplot analysis demonstrated that while median infection levels were moderate, several individuals exhibited exceptionally high egg counts, indicating intense parasitic burden. These findings are of particular concern, as high infection intensity is strongly associated with increased risk of morbidity, including haematuria, bladder pathology, and long-term urogenital complications. The presence of such elevated intensities in specific individuals suggests focal transmission hotspots within these districts, potentially linked to environmental exposure, water contact behaviours, or gaps in preventive chemotherapy coverage. These observations underscore the urgent need to intensify control interventions in Doume and Bertoua, including targeted MDA, improved access to safe water and sanitation, and enhanced health education campaigns. Moreover, the data support the implementation of more frequent monitoring and evaluation to identify and respond to high-risk communities within endemic areas.

The integration of geospatial technologies with parasitological surveillance in this study facilitated the precise identification of high-risk communities and schools, enabling targeted recruitment for FGS screening (Nyotue et al. 2025). This targeted, data-driven approach is recognized as essential for improving diagnostic efficiency and optimizing resource allocation, particularly for neglected manifestations such as FGS that remain chronically underdiagnosed. FGS presents unique challenges at the intersection of gender, health inequity, and neglected tropical diseases. Its clinical diagnosis is hindered by nonspecific symptoms, limited awareness among healthcare providers, and pervasive stigma related to reproductive health, as highlighted by previous studies (Schuster et al. 2022; Williams et al. 2022; Masong et al. 2024). In this context, precision mapping represents a transformative tool for advancing gender-responsive health services by identifying women and girls at greatest risk and facilitating their linkage to care.

Beyond the operational advantages, our study illustrates how precision mapping, coupled with geographic information system (GIS) tools, enhances the spatial resolution of disease surveillance and supports localized, evidence-based public health responses. The integration of electronic data collection and real-time spatial analysis significantly improved the timeliness, accuracy, and granularity of surveillance, which is critical for achieving elimination targets. Emerging technological advances, including the application of machine learning, big data analytics, and remote sensing, hold considerable promise for further refining schistosomiasis risk prediction models (Chen et al. 2024; Xu et al. 2024; Zhou et al. 2024). Such innovations can complement traditional field-based surveys, enhance the predictive power of spatial models, and guide proactive intervention strategies. However, translating these technological advances into measurable public health gains requires sustained investment in capacity building, community engagement, and the integration of geospatial tools within routine health information systems. Furthermore, efforts to tackle FGS must extend beyond clinical diagnosis to address underlying structural drivers, including limited access to water, sanitation, and hygiene (WASH) infrastructure and entrenched gender inequities.

While this study demonstrates the operational feasibility and public health value of precision mapping in identifying high-risk communities for urogenital schistosomiasis and FGS, the findings have important implications for schistosomiasis control policy and programming in Cameroon and other endemic regions. The observed heterogeneity in infection prevalence highlights the limitations of uniform, nationwide mass drug administration strategies and underscores the need for geographically targeted, data-driven interventions. In Bertoua, where prevalence remains particularly high among adolescent girls and young women aged 10–20 years, tailored interventions should be implemented to address the disproportionate risk faced by this vulnerable group. These interventions may include intensified FGS screening, targeted health education, and improved access to reproductive health services. In Doume, where infection appears more uniformly distributed across communities, broader community-wide control strategies are warranted to interrupt transmission at the population level. These strategies should be complemented by sustained investments in water, sanitation, and hygiene (WASH) infrastructure, particularly in high-burden areas where environmental contamination perpetuates the cycle of transmission.

## Conclusion

Precision mapping is a valuable tool for guiding FGS screening in endemic regions. By identifying high-risk communities and infected individuals, it supports targeted interventions, improves diagnostic efficiency and optimizes resource use. Its application in Cameroon has guided FGS screening and improved health outcomes. The approach demonstrated in Cameroon can be scaled to other settings, contributing to the global effort to eliminate schistosomiasis and address gender-specific health disparities.
